# Beta-lactam-induced immediate hypersensitivity reactions: A genome-wide association study of a deeply phenotyped cohort

**DOI:** 10.1016/j.jaci.2020.10.004

**Published:** 2021-05

**Authors:** Paola Nicoletti, Daniel F. Carr, Sarah Barrett, Laurence McEvoy, Peter S. Friedmann, Neil H. Shear, Matthew R. Nelson, Anca M. Chiriac, Natalia Blanca-López, José A. Cornejo-García, Francesco Gaeta, Alla Nakonechna, Maria J. Torres, Cristiano Caruso, Rocco L. Valluzzi, Aris Floratos, Yufeng Shen, Rebecca K. Pavlos, Elizabeth J. Phillips, Pascal Demoly, Antonino Romano, Miguel Blanca, Munir Pirmohamed

**Affiliations:** a Icahn School of Medicine at Mount Sinai, New York, NY; b Sema4, Stamford, Conn; c Department of Molecular and Clinical Pharmacology, University of Liverpool, Liverpool, United Kingdom; d Dermatology Unit, Sir Henry Wellcome Research Laboratories, School of Medicine, University of Southampton, Southampton, United Kingdom; e Division of Dermatology, Department of Medicine, University of Toronto, Toronto, Ontario, Canada; f Deerfield, New York, NY; g Division of Allergy, Hôpital Arnaud de Villeneuve, University Hospital of Montpellier, Montpellier, France; h Infanta Leonor University Hospital, Madrid, Spain; i Allergy Research Group, Allergy Research Group, Instituto de Investigación Biomédica de Málaga-IBIMA, ARADyAL, Malaga, Spain; j Allergy Unit, Columbus Hospital, Fondazione Policlinico Universitario Agostino Gemelli, Istituto di Ricovero e Cura a Carattere Scientifico, Rome, Italy; k Liverpool University Hospitals Foundation National Health Service Trust, Liverpool, United Kingdom; l Allergy Unit, Hospital Regional Universitario de Málaga, Malaga, Spain; m Division of Allergy, University Department of Pediatrics, Pediatric Hospital Bambino Gesù, Rome, Italy; n Department of Systems Biology, New York, NY; o Department of Biomedical Informatics, Columbia University, New York, NY; p Wesfarmers Centre for Vaccines and Infectious Diseases, Telethon Kids Institute, University of Western Australia, Nedlands, Australia; q Department of Medicine, Vanderbilt University Medical Center, Nashville, Tenn; r Centre for Clinical Pharmacology and Infectious Diseases, Institute for Immunology and Infectious Diseases, Murdoch University, Murdoch, Australia; s Unité Mixte de Recherche en Santé (UMR-S) 1136 Institut National de la Santé et de la Recherche Médicale–Sorbonne Université, Equipe Epidemiology of allergic and respiratory diseases (EPAR)– Louis d'Epidémiologie et de Santé Publique (IPLESP), Paris, France; t Istituto di Ricovero e Cura a Carattere Scientifico Oasi Maria SS, Troina, Italy; u Fondazione Mediterranea GB Morgagni, Catania, Italy; v Division of Dermatology, Department of Medicine, Sunnybrook Health Sciences Centre, Toronto, Ontario, Canada

**Keywords:** Type I hypersensitivity, β-lactams, penicillins, cephalosporins, allergy, anaphylaxis, pharmacogenomics, BL, β-Lactam, OR, Odds ratio, QTL, Quantitative trait loci, SNP, Single nucleotide polymorphism

## Abstract

**Background:**

β-lactam antibiotics are associated with a variety of immune-mediated or hypersensitivity reactions, including immediate (type I) reactions mediated by antigen-specific IgE.

**Objective:**

We sought to identify genetic predisposing factors for immediate reactions to β-lactam antibiotics.

**Methods:**

Patients with a clinical history of immediate hypersensitivity reactions to either penicillins or cephalosporins, which were immunologically confirmed, were recruited from allergy clinics. A genome-wide association study was conducted on 662 patients (the discovery cohort) with a diagnosis of immediate hypersensitivity and the main finding was replicated in a cohort of 98 Spanish cases, recruited using the same diagnostic criteria as the discovery cohort.

**Results:**

Genome-wide association study identified rs71542416 within the Class II HLA region as the top hit (*P* = 2 × 10^−14^); this was in linkage disequilibrium with *HLA-DRB1∗10:01* (odds ratio, 2.93; *P* = 5.4 × 10^−7^) and *HLA-DQA1∗01:05* (odds ratio, 2.93, *P* = 5.4 × 10^−7^). Haplotype analysis identified that *HLA-DRB1∗10:01* was a risk factor even without the *HLA-DQA1∗01:05* allele. The association with *HLA-DRB1∗10:01* was replicated in another cohort, with the meta-analysis of the discovery and replication cohorts showing that *HLA-DRB1∗10:01* increased the risk of immediate hypersensitivity at a genome-wide level (odds ratio, 2.96; *P* = 4.1 × 10^−9^). No association with *HLA-DRB1∗10:01* was identified in 268 patients with delayed hypersensitivity reactions to β-lactams.

**Conclusions:**

*HLA-DRB1∗10:01* predisposed to immediate hypersensitivity reactions to penicillins. Further work to identify other predisposing HLA and non-HLA loci is required.

β-Lactam (BL) antibiotics cause a wide spectrum of hypersensitivity reactions (sometimes termed allergy). The self-reported incidence of BL allergy ranges from 1% to >10%,^1^ but in clinic populations, most patients (∼95%) are not found to be truly allergic with validated skin testing and oral challenge. Indeed, a high proportion are intolerant^2^ as adverse effects such as diarrhea after the use of BLs are often mistakenly reported as allergy by patients.

True BL hypersensitivity reactions are classified according to the time of onset of the reaction following drug intake.^3^ Immediate hypersensitivity reactions develop in minutes or hours after drug intake and are due to cross-linking of specific IgE molecules on the mast cell surface with release of vasoactive mediators such as histamine leading to vasodilation, increased vascular permeability, and smooth muscle contraction.^4^ Clinically this is manifested as urticaria, angioedema, bronchospasm, and hypotension. Anaphylaxis is the most severe and feared form of immediate hypersensitivity. By contrast, delayed hypersensitivity reactions occurring >6 hours after dosing are typically T-cell-mediated and have variable manifestations including maculopapular exanthem, drug reaction with eosinophilia and systemic symptoms, and Stevens-Johnson syndrome/toxic epidermal necrolysis.^3^

Medicines are among the main cause of fatal anaphylaxis with a mortality rate higher than with other agents.^5^ Penicillins and cephalosporins are still the 2 most common drug classes associated with anaphylaxis,^6^ with penicillins having a higher incidence (1-5 per 100,000)^7^ compared with cephalosporins.^1^ Cross-reactivity between penicillins, cephalosporins, and other BLs not sharing an R1 or R2 side chain is now thought to be <2%.^8^^,^^9^

Potential clinical risk factors for immediate hypersensitivity reactions are family history, atopy, concomitant virus infections, and the route of administration.^10^ Genetic predisposing factors have also been identified:^10^ the most comprehensive was an analysis of 107,398 single nucleotide polymorphisms (SNPs) that identified that the *HLA-DRA* locus may protect against penicillin-induced immediate hypersensitivity reactions.^11^ To further investigate the role of genetic factors in BL-induced immediate hypersensitivity reactions, we have undertaken a genome-wide association study of the largest deeply phenotyped patient cohort assembled so far.

## Methods

### Cases

All subjects were recruited between 2009 and 2013 as part of International Consortium on Drug Hypersensitivity, involving 5 recruitment centers worldwide (Australia, France, Italy, Spain, and United Kingdom). The study was approved by ethics committees in all countries, and all patients gave written informed consent.

We recruited 662 patients (the discovery cohort) with a diagnosis of immediate hypersensitivity to BL antibiotics (Table I). The diagnosis of immediate hypersensitivity was made in specialist allergy clinics, as per published criteria.^12^ All patients required immunological assessment (total and specific IgE, skin testing including skin prick test and intradermal and/or oral provocation) as part of the inclusion criteria. Independent adjudication of all cases was undertaken by N.H.S. and P.S.F. For replication of any signals, we separately recruited another 98 patients with immediate hypersensitivity from a clinic in Spain, diagnosed according to the same criteria.

**Table I:** Causative drugs and clinical variables broken down across the discovery and replication cohorts

Clinical characteristics	Immediate hypersensitivity		Delayed hypersensitivity cohort (n = 268)
	Discovery cohort (n = 662)	Replication cohort (n = 98)	
Female, n (%)	416 (62)	56 (57)	174 (64)
Age (y) mean ± SD (% missing)	42.0 ± 16 (27)	51.4 ± 12.3 (0)	44.5 ± 20 (73)
History of allergies, % (no. with available information)	31 (658)	9 (98)	30.6 (268)
No. of ADRs, mean ± SD (no. with available information)	1.1 ± 0.3 (659)	1.2 ± 0.5 (98)	1 ± 0.2 (251)
Autoimmune disease diagnosis, %	9	6	7
Positive skin test, % (total no. tested)	85 (578)	78 (67)	93 (204)
Positive prick test, % (total no. tested)	45 (142)	37 (82)	82 (207)
Positive oral provocation/rechallenge, % (total no. tested)	76 (106)	65 (20)	94 (17)
Clinical symptoms, n (%)			
Immediate hypersensitivity manifestations∗	662 (100)	98 (100)	—
AGEP	—	—	14 (5)
DRESS	—	—	7 (3)
Mild reactions including maculopapular exanthem	—	—	212 (79)
SJS/TEN	—	—	36 (13)
Drug class, n (%)			
Penicillin	501 (75)	98 (100)	246 (92)
Cephalosporin	162 (25)	—	20 (7.5)
Other BLs	—	—	2 (0.01)
Suspected causal drug			
Amoxicillin	165 (25)	65 (66)	77 (29)
Ampicillin	36 (5)	—	54 (20)
Bacampicillin	20 (3)	—	21 (8)
Cefaclor	23 (3)	—	—
Cefazolin	17 (3)	—	4 (1.5)
Cefotaxime	17 (3)	—	—
Ceftazidime	18 (3)	—	1 (0.4)
Ceftriaxone	52 (8)	—	4 (1.5)
Cefuroxime	14 (3)	—	2 (0.7)
Co-amoxiclav	218 (33)	26 (26)	70 (26)
Phenoxymethylpenicillin	24 (4)	7 (7)	5 (2)
Piperacillin	18 (3)	—	5 (2)
Other	41 (6)	—	25 (9)

*ADRs*, Adverse drug reactions; *AGEP*, acute generalized exanthematous pustulosis; *DRESS*, drug reaction with eosinophilia and systemic symptoms; *SD*, standard deviation; *SJS/TEN,* Stevens-Johnson syndrome/toxic epidermal necrolysis.

∗ See text for nature of clinical manifestations.

To determine specificity of any signals identified in patients with immediate hypersensitivity, we also evaluated 268 patients with delayed hypersensitivity reactions across multiple BLs. The diagnosis again was in accordance with published guidance,^12^ and all cases were adjudicated by N.H.S. and P.S.F. We also included an additional 17 BL-induced delayed hypersensitivity reaction cases analyzed in Shen et al.^13^

### Controls

We used general population samples as study controls. This comprised 9217 European ancestry controls from multiple available sources enriching the group with Spanish, Italian, and north European origin samples because cases were mainly recruited from those countries. We used the Wellcome Trust Case Control Consortium (http://www.wtccc.org.uk); the Population Reference Sample (POPRES),^14^ PGX4000119,^13^ LAM30004,^13^ and Spanish Bladder cancer cohort (phs000346.v1)^15^ from the Database of Genotypes and Phenotypes (dbGaP); Hypergenes cohort (http://www.hypergenes.eu/); the National Spanish DNA Bank (http://www.bancoadn.org/); and Toscani in Italia ([TSI] HapMap data) to obtain ancestry control data. In addition, we also recruited a group of 137 penicillin-tolerant controls from Italy.

### Genotyping

Genome-wide genotyping of DNA extracted from whole blood was performed at the Broad Institute (Boston, Mass) for 662 cases with BL-induced immediate hypersensitivity and 268 cases with delayed hypersensitivity reaction, and from 137 penicillin-tolerant controls from Italy. In 474 (354 BL-induced immediate and 120 BL-induced delayed) cases, the Illumina Infinium HumanCoreExome BeadChip (Illumina, Inc, San Diego, Calif) was used while for 439 (308 BL-induced immediate and 131 BL-induced delayed) cases, the Illumina HumanOmniExpress BeadChip was used. In this last batch, we also genotyped 137 Italian penicillin-tolerant controls. In addition, the BL-induced delayed case group also included 17 BL-delayed hypersensitivity cases previously genotyped by the Illumina 1M Duo chip, extracted from a larger Stevens-Johnson syndrome/toxic epidermal necrolysis study that included multiple drugs, as described by Shen et al.^13^ Other control cohorts were publicly available (see Table E1 in this article’s Online Repository at www.jacionline.org). For each of the genotyping cohort, standard quality control was conducted at both single marker and subject levels as previously described.^13^ This was followed by SNP and HLA imputation and amino acid analysis (see the Methods section in this article’s Online Repository at www.jacionline.org).

### Replication cohort SNP and HLA genotyping

The top associated imputed SNPs were validated by SNP genotyping using either TaqMan, SNP genotyping assays (Thermo Fisher Scientific, Paisley, UK) or iPLEX MassArray genotyping platform (Agena Biosciences, Hamburg, Germany). High-resolution genotyping of *HLA-A, HLA-B, HLA-C, DRB1, DQA1,* and *DQB1* was performed by Histogenetics (Ossining, NY). Sequencing data files were analyzed using Histogenetics’ proprietary analysis software (Histomatcher and HistoMagic) for HLA genotype calling. Allele assignments are based on IMGT/HLA Database (release version 2.21.0, dated April 2008; http://www.ebi.ac.uk/imgt/hla/).

### Statistical analysis

The effect of population structure was assessed through principal component analysis using the smartPCA program from the EIGENSTRAT package (version 3.0; Alkes Price, Harvard T.H. Chan School, Boston, Mass).^16^ Single marker and haplotype association analyses and heterogeneity test analyses were carried out by PLINK 1.07.^17^ The statistical association of each marker, HLA alleles and SNPs, was determined in a logistic regression framework with scores for the first 7 principal components as covariates under an additive model using PLINK. We used the same statistical test for subpopulation analyses, using the 2, 7, and 10 most significant principal components as covariates in Italian, Spanish, and North European populations, respectively. We set the genome-wide traditional significance P-value threshold to 5.0 × 10^−8^ to correct for multiple testing and MHC-wide significance threshold to 2.0 × 10^−4^ to correct for total number of predicted alleles. When we obtained genome-wide significant signals, we tested for independent effects from the neighboring variants by including the most associated variants as a covariate and then testing the significance of others in the region. All detailed analyses and Manhattan plots were performed with R (version 3.0.2; R Foundation, Vienna, Austria). Regional plots were drawn by LocusZoom.^18^ Meta-analysis was performed using a fixed-effect model in the *metafor* package (http://www.metafor-project.org/doku.php/metafor).

## Results

### Patient cohorts

The clinical characteristics of the patients are shown in Table I. Clinical manifestations in the discovery cohort included angioedema (35%), bronchospasm (24%), and urticaria (34%), while hypotension was reported in only 4% of cases. The length of reaction in patients with immediate hypersensitivity was 2 to 11 days, while it ranged from 21 to 26 days for patients with delayed hypersensitivity reactions. Patients were included if they had positive diagnostic assessment, as highlighted in Table I. Penicillins accounted for 75% of cases, with the most common culprit drug being amoxicillin accounting for 58% of cases in the discovery cohort.

### Association with immediate reactions to BLs

**Fig E1:**
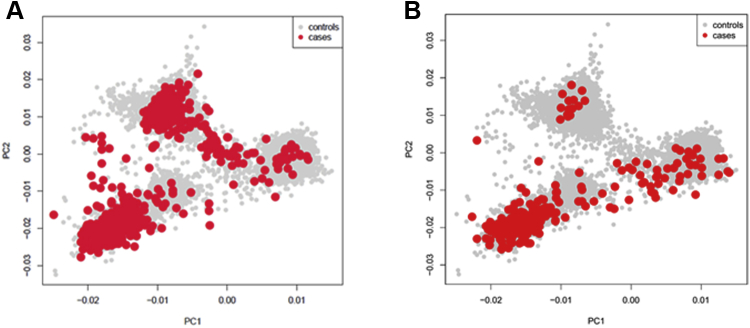
Scatterplot representing the first 2 principal components *(PCs)* of the current study cohorts, cases and controls: BL-immediate hypersensitivity reactions **(A)** and BL-delayed hypersensitive reactions **(B)**.

We first conducted a genome-wide association study on 662 patients of European descent with immediate hypersensitivity reactions and 9217 previously genotyped population controls matched for ethnicity. The total number of SNPs, which were included in the analyses after quality control, was 4,265,742. The cases clustered within 3 major groups (Italian, Spanish, and Northern European) (see Fig E1 in this article’s Online Repository at www.jacionline.org) in keeping with the self-reported ethnicity.

**Fig 1:**
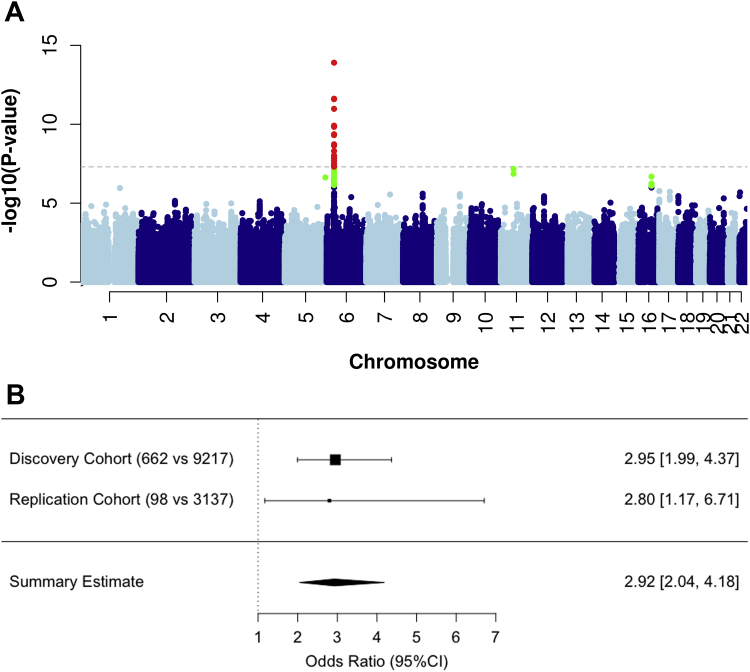
Genomic data in patients with immediate hypersensitivity reactions. **A,** Manhattan plot displaying the association analysis undertaken in patients with immediate hypersensitivity reactions to BLs (n = 662). SNPs in green have a significance level <5 × 10^−6^ and red have a significance level <5 × 10^−8^. **B,** Forest plot showing the effect size of the association between *HLA-DRB1∗10:01* and immediate reactions in the discovery and replication cohorts. For each analysis, the OR of the association is reported with 95% CI. The dimension of the squares is proportional of the number of cases.

**Fig E2:**
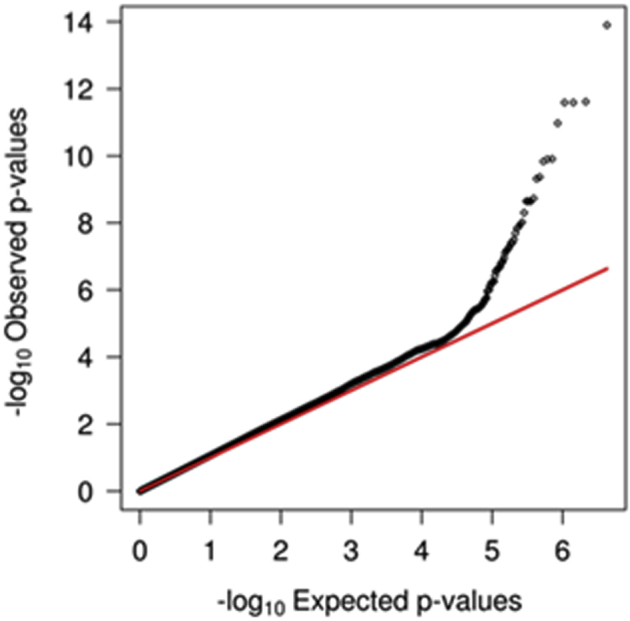
The figure shows the QQ plot for the current analysis. The inflation factor is 1.09.

A genome-wide significant association was identified within the Class II HLA region, rs71542416 being the top hit (odds ratio [OR], 5.17; 95% CI, 3.40-5.17; *P* = 2 × 10^−14^) (Table II, Fig 1, *A*, and see Fig E2 in this article’s Online Repository at www.jacionline.org). The frequency of rs71542416 in our control population was comparable with that reported in publicly available sources (Table II). HLA allele imputation using HLA genotype imputation with attribute bagging, or HIBAG,^19^ showed the *HLA-DRB1∗10:01* (OR, 2.95; 95% CI, 1.99-4.36; *P* = 6.0 × 10^−8^) and *HLA-DQA1∗01:05* (OR, 2.93; 95% CI, 1.92-4.45; *P* = 5.4 × 10^−7^) alleles to be significantly associated with the immediate reactions, with consistent ORs (Table II) and were tagged by rs71542416 (*r*^2^ = 0.76). Haplotype analysis identified that *HLA-DRB1∗10:01* was a risk factor even without the *HLA-DQA1∗01:05* allele (see Table E2 in this article’s Online Repository at www.jacionline.org). *HLA-DRB1∗10:01* was seen in 3% of cases and <1% of controls. The frequency of the HLA alleles within the Italian penicillin-tolerant controls was 10 times less than in the Italian general population (0.1% vs 1%).

**Table II:** The most significantly associated variants for immediate hypersensitivity reactions to BLs

	Minor allele frequency			Association analysis		Association conditioned for HLA haplotype∗		Association conditioned for rs71542416	
	Cases	Controls	Population reference cohort	OR (95% CI)	*P* value	OR (95% CI)	*P* value	OR (95% CI)	*P* value
*HLA-DRB1∗10:01*	0.03	0.008	0.008	2.95 (1.99-4.36)	6.0 × 10^−8^	—	—	0.60 (90.19-1.85)	.37
*HLA-DQA1∗01:05*	0.03	0.01	0.01	2.93 (1.92-4.46)	5.4 × 10^−7^	—	—	0.79 (0.32-1.91)	.60
rs71542416	0.03	0.006	0.008	5.17 (3.40-5.17)	1.2 × 10^−14^	8.22 (2.68-25.23)	.0002	—	—
rs114632839†	0.25	0.367	0.40	0.77 (0.67-0.89)	.0003	0.69 (0.60-0.80)	1.1 × 10^−6^	0.68 (0.59-0.79)	6.1 × 10^−7^

Minor allele frequency for external data obtained from allelefrequncy.net for HLA alleles or GnomAD for SNPs. ORs are of the logistic regression model, correcting for population stratification. *P* values are logistic regression *P*.

∗ HLA haplotype was HLA-DRB1∗10:01–HLA-DQA1∗01:05.

† The marker rs114632839 has merged with rs3135392.

The HLA allele effect size was similar across the 3 major clusters (heterogeneity test, *P* = .11) (Table III). The positive predictions in cases were fully validated by direct HLA typing. An additional 67 cases with low-quality predictions in both the loci were also typed. Among them, we found only 1 positive carrier for *HLA-DRB1∗10:01*. All cases were also genotyped for rs71542416—this showed a concordance of 99% between typed and imputed genotypes of rs71542416. *HLA-DRB1∗10:01* co-occurred with rs71542416 in 89% of the *HLA-DRB1∗10:01*–positive patients, while 12% of all cases carried rs71542416 alone.

**Table III:** The association between *HLA-DRB1∗10:01* and rs71542416, and BL-induced immediate hypersensitivity reactions across the different nationalities

Ethnic cluster	No. of cases∗	Minor allele frequency		OR (95% CI)	*P* value
		Cases	Controls		
*HLA-DRB1∗10:01*					
Italians	352	0.021	0.012	2.33 (1.15-4.73)	.02
Spanish	226	0.049	0.014	3.81 (2.27-6.42)	4.74 × 10^−7^
Northern Europeans	61	0.025	0.004	3.93 (1.17-13.21)	.03
rs71542416					
Italians	352	0.02	0.007	4.33 (1.98-9.49)	.0002
Spanish	226	0.05	0.008	6.80 (3.89-11.87)	1.69 × 10^−11^
Northern Europeans	61	0.02	0.004	4.42 (1.29-15.13)	.02

OR and *P* value as defined in Table II.

∗ Numbers represent homogeneous populations within clusters after principal component analysis.

**Fig E3:**
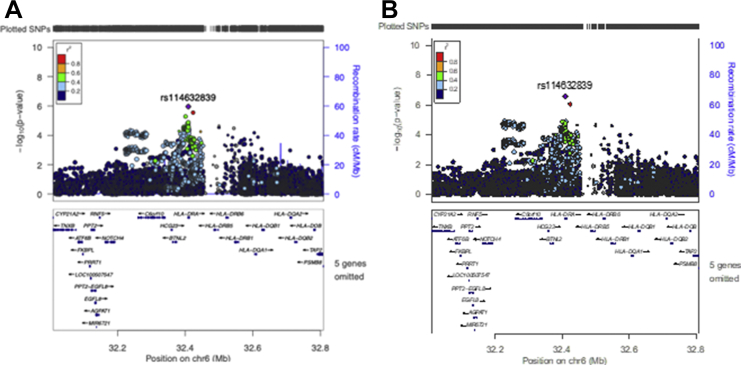
Regional plots of the MHC region for the conditioned analysis on HLA-*DRB1∗10:01/HLA-DQA1∗01:05* haplotype **(A)** and on rs71542416, the most associated SNP **(B)**.

**Fig E4:**
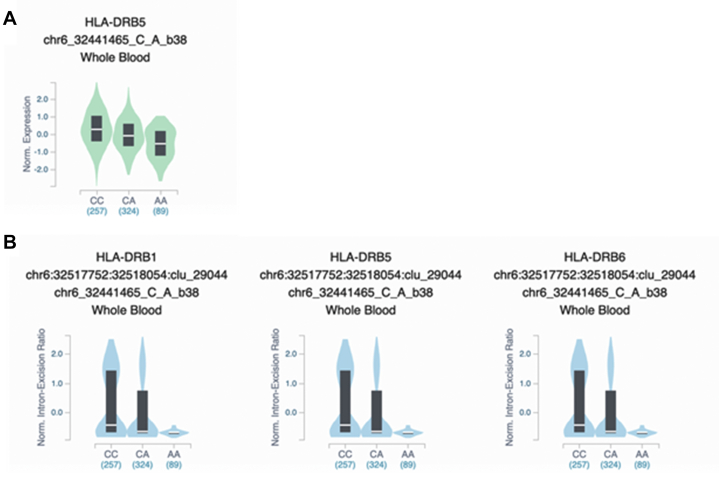
Violin plots from the GTEx website for expression QTL **(A)** and splicing QTL **(B)** effects of rs114632839 in whole blood.

Including rs71542416 or the HLA alleles as covariates revealed a residual protective effect of the *HLA-DRA* locus, tagged by rs114632839, an intronic gene variant, in accordance with the findings of Gueant et al^11^ (Table II and see Figs E3 and E4, *A* in this article’s Online Repository at www.jacionline.org). Interestingly Genotype-Tissue Expression Program, or GTEx (Common Fund, National Institutes of Health, Bethesda, Md), analysis revealed that this variant was a strong expression quantitative trait loci (QTL) for *HLA-DRB5* (*P* = 5.3 × 10^−23^) and splicing QTL for the *HLA-DRB1* (*P* = 1.1 × 10^−16^), *HLA-DRB5* (*P* = 1.1 × 10^−16^) and *HLA-DRB6* (*P* = 1.1 × 10^−16^) loci with the minor alleles showing a lower intron excision ratio. Both effects were detected in whole blood and shared across other tissues (Fig E4, *B*).

**Fig E5:**
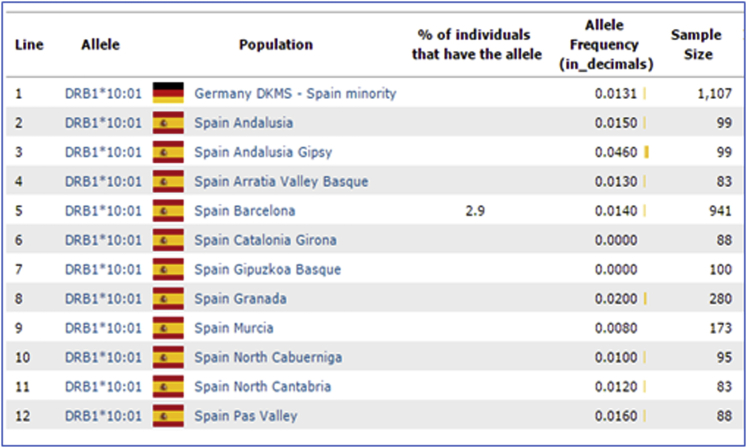
Spanish studies reported on allelefrequency.net used as replication control set.

A replication cohort of 98 patients with anaphylaxis induced by either amoxicillin or amoxicillin-clavulanate (Table I) was recruited separately from Spain. We identified 7 individuals who were positive for *HLA-DRB1∗10:01*, as confirmed by HLA typing. Comparison using the 11 Spanish HLA-typed cohorts reported at allelefrequency.net provided a total of 3137 Spanish subjects (see Fig E5 in this article’s Online Repository at www.jacionline.org) as ethnically matched population controls. This analysis replicated the association with an OR of 2.80 (95% CI, 1.17-6.71; Fisher exact test, *P* = .016) (Fig 1, *B*).

Meta-analysis of the discovery and replication cohorts showed that *HLA-DRB1∗10:01* increased the risk of immediate hypersensitivity at a genome-wide level (OR, 2.96; 95% CI, 1.99-4.37; *P* = 4.1 × 10^−9^) (Fig 1, *B*). The sensitivity and specificity of the allele is 0.06 and 0.98, respectively, while the positive and negative predictive values are 17% and 94%, respectively.

The most significantly associated amino acid with immediate hypersensitivity reactions was glutamate at position 10 (OR, 2.72; 95% CI, 1.81-4.08; *P* = 1.4 × 10^−6^) (see Table E3 in this article’s Online Repository at www.jacionline.org). Amoxicillin, amoxicillin-clavulanic acid, and phenoxymethylpenicillin showed the highest effect size (see Table E4 in this article’s Online Repository at www.jacionline.org). Glutamate-10 co-occurred with other amino acids (arginine-30, valine-31, alanine-38, tyrosine-40, proline-231, glutamine-166) that had the same frequency in cases and controls as glutamate-10 and *HLA-DRB1∗10:01* (Table E3). However, association with these amino acids disappeared after condition for either glutamate-10 or *HLA-DRB1∗10:01* (Table E3). Interestingly, glutamate-10 co-occurred with the shared epitope RRA at positions 70, 71 and 74, previously associated with seropositive rheumatoid arthritis^20^ and specific for the *HLA-DRB1∗10:01* allele. The ERRA haplotype increased risk (OR, 2.72; *P* = 1.4 × 10^−6^) equivalent to that seen with glutamate-10 alone. None of the other risk/protective amino acid motifs for seropositive rheumatoid arthritis^20^ spanning positions 70 to 74 in the *DRB1* locus (such as QRRAA risk motif or DERAA and DRRAA protective motifs) were associated with our phenotype.

### HLA analysis in patients with delayed hypersensitivity reactions

To determine whether the association with *HLA-DRB1∗10:01* was limited to patients with immediate hypersensitivity reactions, we analyzed 268 patients with delayed hypersensitivity to a variety of BLs (Table I) using the same control set (Fig E1, *B*). No association was identified for *HLA-DRB1∗10:01* (n = 249; OR, 1.34; 95% CI, 0.55-3.26; *P* = .5).

### Drug-specific associations with immediate hypersensitivity

*HLA-DRB1∗10:01* was associated with penicillins as a class (OR, 3.07), but not with cephalosporins (Table IV). Among the penicillins, the strongest signals were for amoxicillin (OR, 3.48), amoxicillin clavulanic acid (OR, 2.85), and phenoxymethylpenicillin (OR, 6.66) (Table IV). When we combined amoxicillin and amoxicillin clavulanic acid cases (assuming that amoxicillin rather than clavulanic acid was the culprit), the OR was 3.1 (95% CI, 2.01-4.85; *P* = 4.0 × 10^−7^). Additional drug-specific HLA allele associations that we identified will need confirmation (see Tables E5 and E6 in this article’s Online Repository at www.jacionline.org).

**Table IV:** Effect size of the association of *HLA-DRB1∗10:01* with immediate hypersensitivity reactions broken down by drug classes and individual drugs

Drug	Ethnicity∗	No. of cases	Case MAF	OR (95% CI)	*P* value
Cephalosporins	Caucasian	162	0.019	2.03 (0.82-5.07)	.13
Cefaclor	Caucasian	23	0	—	—
Cefazolin	Caucasian	17	0.059	6.12 (1.32-28.30)	.02
Cefotaxime	Italian	17	0	—	—
Ceftazidime	Italian	17	0	—	—
Ceftriaxone	Italian	48	0.010	1.05 (0.13-8.33)	.96
Cefuroxime	Caucasian	14	0.050	2.90 (0.37-22.76)	.31
Penicillins	Caucasian	501	0.036	3.07 (2.04-4.62)	7.42 × 10^−8^
Amoxicillin	Caucasian	166	0.042	3.48 (1.92-6,28)	3.74 × 10^−5^
Ampicillin	Italian	29	0.014	1.98 (0.25-15.79)	.52
Co-amoxiclav	Caucasian	218	0.034	2.85 (1.60-5.10)	.0004
Phenoxymethylpenicillin	Caucasian	25	0.080	6.66 (2.14-20.79)	.001
Piperacillin	Caucasian	18	0.028	2.32 (0.29-18.78)	.43
Bacampicillin	Italian	21	0.024	2.09 (0.26-17.03)	.49

*MAF*, Minor allele frequency. OR and *P* value as defined in Table II.

∗ Ethnicity—Caucasian is applied to patients of Spanish, Italian, and Northern European descent and confirmed by principal component analysis. Where only 1 nationality was available for a particular drug, this is indicated and only appropriate matching controls were chosen.

**Fig E6:**
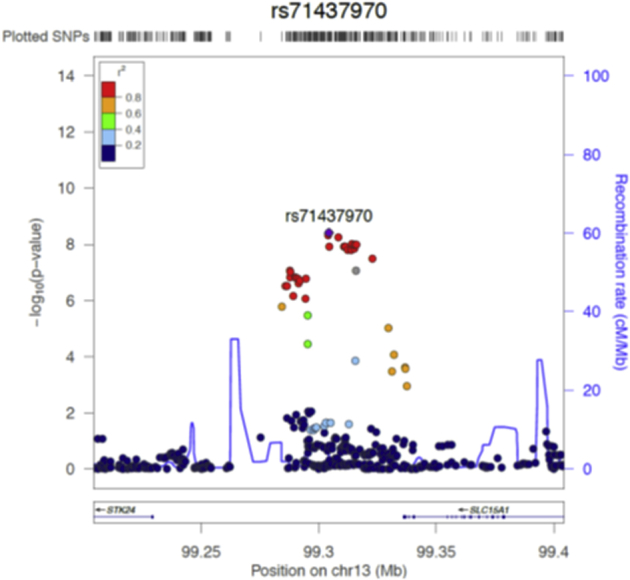
Regional plot of the chromosome 13 signal associated with amoxicillin-induced immediate hypersensitivity in the discovery cohort. The horizontal axis shows SNPs along the chromosomal region and the vertical axis shows the (−log10 transformed) observed *P* value. SNPs are color-coded to depict their linkage disequilibrium (LD) with the top SNP, shown in purple. Estimated recombination rates are plotted on the right vertical axis in cyan to reflect the local LD structure.

In the drug-specific analysis, a genome-wide signal (rs71437970) on chromosome 13 upstream of *SLC15A1* (see Fig E6 in this article’s Online Repository at www.jacionline.org) was identified for the amoxicillin cases (OR, 2.94; *P* = 3.8 × 10^−9^) (Table E6). This association was shared across the European subpopulations and with amoxicillin-clavulanate cases (see Tables E7 and E8 in this article’s Online Repository at www.jacionline.org). However, we failed to replicate the association, with an allele frequency that was lower than that observed in Spanish controls (0.007 vs 0.04).

## Discussion

We have identified an association between the SNP rs71542416 and immediate hypersensitivity reactions to penicillins. The SNP does not affect gene expression in GTEx but is in linkage disequilibrium with *HLA-DRB1∗10:01* and *HLA-DQA1∗01:05.* Haplotype analysis identified that *HLA-DRB1∗10:01* was a risk factor even without the *HLA-DQA1∗01:05* allele suggesting that *HLA-DRB1∗10:01* may be the predominant driver of the association. However, 12% of cases carried rs71542416 but were negative for *HLA-DRB1∗10:01,* suggesting that the SNP may be a tag for other rare HLA alleles, which is consistent with the hypothesis of Heap et al^21^ who showed an association between *HLA-DQA1-HLA-DRB1* variants and thiopurine-induced pancreatitis.

The association with *HLA-DRB1∗10:01* and rs71542416 was most pronounced in the Spanish cohort (Table III), but given that the ORs were of similar magnitude in all populations studied, there was overlap in the CIs, and the prevalence of the SNP and HLA allele, our findings can be generalized across the European subethnicities studied (Table III). However, further studies will be needed in both European and non-European populations to determine the global relevance of this association. Additionally, the association was limited to immediate reactions and was not observed with the delayed hypersensitivity reactions, highlighting the specific nature of the association. Evaluation of drug-specificity showed associations with amoxicillin, amoxicillin-clavulanate, and phenoxymethylpenicillin. However, given the limited sample size with the other penicillins, we cannot exclude the possibility of an association with all penicillins (Table IV). Similarly, we did not find an association with cephalosporins, but this may also be because of a lower sample size.

The clear strength of our study is that all patients were deeply phenotyped: there was a clear clinical history with a temporal relationship to drug intake, and the diagnosis was confirmed immunologically by skin testing and/or oral provocation. Such deep phenotyping is important because many patients claim to be allergic to penicillin, but very few are; of those claiming to be allergic, <1 in 20 have an acute reaction to an oral challenge (the gold standard clinical test to confirm an IgE-mediated reaction).^22^

Our data add to the increasing evidence of HLA in predisposing to different clinical phenotypes of drug hypersensitivity reactions.^23^ The most well-known of these associations is *HLA-B∗57:01* and abacavir hypersensitivity,^24^ which has been implemented into clinical practice and has resulted in a significant reduction in abacavir hypersensitivity.^25^ It is important to note that most of the HLA associations identified to date have been with delayed hypersensitivity reactions.^23^ However, more recent studies have identified HLA alleles as predisposing factors for immediate reactions. For instance, *HLA-DRB1∗07:01* is a risk factor for the development of anti-asparaginase antibodies and immediate reactions.^26^ Our data, which show that *HLA-DRB1∗10:01* predisposes to immediate hypersensitivity, are also consistent with the pathogenesis of immediate reactions where the interaction between B cells and CD4^+^/T_H_2-positive cells, through HLA Class II alleles, is central to the immunoglobulin switching that leads to the generation of specific IgE antibodies. Different HLA alleles have been associated with other types of immune-mediated reactions caused by BLs. For example, *HLA-B∗57:01* predisposes to flucloxacillin-induced cholestatic hepatitis,^27^ while liver injury caused by amoxicillin-clavulanate is associated with the Class II HLA haplotype *HLA-DRB1∗1501-DQB1∗0602.*^28^ Mechanistic studies undertaken in our laboratory have shown that drug-specific, HLA-restricted T cells can be isolated from patients with a past history of liver injury due to flucloxacillin^29^ and amoxicillin-clavulanate.^30^ It will be valuable to conduct similar studies in patients with a history of penicillin-induced immediate reactions to understand the mechanistic basis of the association with *HLA-DRB1∗10:01.*

Another potentially interesting finding in this study was the association between *SLC15A1* gene variants and amoxicillin-induced immediate reactions. SLC15A1 encodes the human peptide transporter 1, which is known to transport amoxicillin.^31^ Therefore, it is plausible that variation in the activity of human peptide transporter 1 could result in altered amoxicillin pharmacokinetics and thereby increase risk of a type I reaction. However, we were not able to replicate this finding, and further work (including functional studies) to understand whether this gene is important in predisposing to immediate reactions will be required.

What are the clinical implications of this finding? Given the rarity of penicillin-induced anaphylaxis, the low population prevalence and sensitivity of *HLA-DRB1∗10:01,* and the very wide usage of penicillins, the prospective use of this allele in screening patients before penicillin prescription would not be practical or feasible in terms of both the high numbers needed to test to prevent 1 case and patients unnecessarily excluded from therapy. However, this association of immediate penicillin hypersensitivity with *HLA-DRB1∗10:01* may provide much novel insights into the mechanisms of immediate reactions associated with penicillins, including the mechanisms of sensitization and natural loss or waning of penicillin, which is known to occur over time. Moreover, the higher negative predictive value of the allele (94%) may be of use in risk stratifying patients where penicillin cannot be excluded as an etiological agent in the setting of an immediate reaction.

Our study has limitations. First, the overall sample size is small compared with that used in complex diseases, but it is larger than that used in many pharmacogenomic studies. Our efforts to identify deeply phenotyped patients in this study was a result of an extensive international collaboration highlighting the difficulties in achieving large sample sizes in pharmacogenomic studies. Furthermore, we were unable to perform permutation testing to validate the replication *P* value for *HLA-DRB1∗10:01.* Second, because we used population controls, we could not adjust for self-reported ethnicity, but this is unlikely to have had a major impact as we accounted for this through an analysis of population stratification (Fig E1). Third, matching cases and controls for age, sex, and other comorbidities was not possible because of the use of population controls, and because sex could not be determined due to the absence of X chromosome SNP data. Whether this impacts on the association with the genetic signals identified by us will require further study.

In summary, we have for the first time reported an association of *HLA-DRB1∗10:01* carriage in deeply immunologically phenotyped European ancestry individuals with penicillin-induced immediate type I hypersensitivity reactions. However, we cannot exclude the possibility of other HLA alleles or HLA haplotypes also being important in conferring susceptibility in some patients, and therefore further work in both European and non-European patients is required to identify other HLA alleles, and also whether *HLA-DRB1∗10:01* is universally important. It is also interesting to note that we also identified that rs114632839, which is a proxy for the *HLA-DRA* locus, protected against the development of immediate hypersensitivity reactions to BLs, consistent with a previous study.^11^ rs114632839 is an expression QTL and splicing QTL for several HLA loci, suggesting that predisposition to immediate hypersensitivity to penicillins is likely to be complex and mediated by a combination of susceptibility and protective HLA and non-HLA alleles. Clearly we have reported associations, and proof of causality will require a full understanding of the immunopathogenesis of initial sensitization to penicillin, and in particular, the mechanism of antigen presentation (including the relative importance of the BL ring vs the side chains) and interaction with CD4^+^ T cells that ultimately leads to IgE-switching and the generation of hapten-specific IgE antibodies.

**Clinical implications**

This novel insight into the mechanisms of immediate reactions associated with penicillins may be of use in risk stratifying patients where penicillin cannot be excluded as an etiological agent.

## Figures and Tables

**Table E1:** Genotyping details of the control cohorts

Cohort	No. of samples	Genotyping Array
WTCCC	4824	Illumina 1M BeadChip
phs000346.v1 (dbGaP)	2072	Illumina 1M BeadChip
Hypergenes	901	Illumina 1M BeadChip
LAM30004 collection	60	Illumina 1M BeadChip
National Spanish DNA Bank	173	Illumina 1M Duo/Illumina Infinium HumanCoreExome BeadChip
	222	Illumina Infinium HumanCoreExome BeadChip
iSAEC Italian penicillin-tolerant controls	137	Illumina HumanOmniExpress BeadChip
PGX40001 collection	85	Illumina 1M Duo BeadChip
TSI	88	Illumina 1M Duo BeadChip
POPRES	655	Illumina 1M Duo BeadChip

*iSAEC,* International Serious Adverse Events Consortium; *WTCCC,* Welcome Trust Case Control Consortium.

**Table E2:** Effect size for the association with the *HLA-DRB1∗10:01* and *HLA-DQA1∗01:05* haplotypes

*HLA-DRB1∗10:01*	*HLA-DQA1∗01:05*	MAF		OR	*P* value
		Cases	Controls		
P	P	0.03	0.01	2.93	.000001
P	A	0.005	0.001	2.84	.04

*A*, Absence of allele in the haplotype; *P*, presence of allele in the haplotype. OR and *P* value as defined in Table II. No individuals carried *HLA-DQA1∗01:05* alone.

**Table E3:** Most significant association results for amino acids in HLA molecules and risk of immediate hypersensitivity to penicillins

Marker		MAF		Association analysis		Association conditioned for glutamate-10		Association conditioned for HLA haplotype	
Position	AA	Cases	Controls	OR (95% CI)	*P* value	OR (95% CI)	*P* value	OR (95% CI)	*P* value
10	E	0.03	0.008	2.72 (1.81-4.09)	.000001	—	—	—	—
30	R	0.03	0.008	2.67 (1.78-4.01)	.000002	—	—	—	—
31	V	0.03	0.008	2.67 (1.78-4.01)	.000002	—	—	—	—
38	A	0.03	0.008	2.67 (1.78-4.01)	.000002	—	—	—	—
40	Y	0.03	0.008	2.67 (1.78-4.01)	.000002	—	—	—	—
231	P	0.03	0.008	2.53 (1.68-3.8)	.000008	—	—	0.4 (0.04-4.2)	.44
166	Q	0.03	0.008	2.52 (1.68-3.79)	.000009	—	—	—	—
11	V	0.17	0.15	1.42 (1.19-1.69)	.00008	1.28 (1.06-1.55)	.008	1.29 (1.07-1.55)	.008
37	Y	0.41	0.3	1.27 (1.11-1.44)	.0004	1.21 (1.06-1.38)	.005	1.21 (1.06-1.38)	.005

**Table E4:** Association between glutamate at position 10 in the *HLA-DRB1∗10:01* molecule and risk of immediate hypersensitivity stratified by class of BL and individual molecules

Drug	Ethnicity	No. of cases	Case MAF	OR (95% CI)	*P* value
Cephalosporins	Caucasian	162	0.01	1.61 (0.59-4.33)	.34
Cefaclor	Caucasian	23	—	—	—
Cefazolin	Caucasian	17	0.06	6.26(1.35-29.98)	.01
Cefotaxime	Italian	17	—	—	—
Ceftazidime	Italian	17	—	—	—
Ceftriaxone	Italian	48	—	—	—
Cefuroxime	Caucasian	14	0.04	2.73 (0.34-21.69)	.34
Penicillins	Caucasian	501	0.03	2.85 (1.85-4.35)	1.2 × 10^−6^
Amoxicillin	Caucasian	166	0.04	3.31 (.80-6.10)	.0001
Ampicillin	Italian	29	0.01	1.88 (0.23-6.10)	.54
Co-amoxiclav	Caucasian	218	0.03	2.44 (1.31-4.51)	.004
Phenoxymethylpenicillin	Caucasian	25	0.08	7.35 (2.37-22.95)	.0005
Piperacillin	Caucasian	18	0.03	2.17 (0.26-17.78)	.46
Bacampicillin	Italian	21	0.02	1.98 (0.24-16.10)	.52

**Table E5:** Effect size of the association of the most significant HLA risk alleles in the amoxicillin clavulanic acid analysis

	MAF		Association analysis		Association conditioned for HLA haplotype	
	Cases	Controls	OR (95% CI)	*P* value	OR (95% CI)	*P* value
*HLA-DRB1∗01:03*	0.02	0.007	5.21 (2.45-11.09)	1.8 × 10^−5^	5.45 (2.56-11.61)	1.1 × 10^−5^
*HLA-DQA1∗01:05*	0.03	0.01	3.42 (1.90-6.15)	4.0 × 10^−5^	—	—
*HLA-DPB1∗13:01*	0.06	0.02	2.24 (1.47-3.42)	1.8 × 10^−4^	2.28 (1.49-3.48)	1.4 × 10^−4^
*HLA-DRB1∗10:01*	0.03	0.008	2.85 (1.60-5.10)	3.9 × 10^−4^	—	—

OR and *P* value as defined in Table II.

**Table E6:** Effect size of *HLA-DRB1∗01:03* and amoxicillin clavulanic acid–induced immediate hypersensitivity reactions across the different ethnic clusters

	No. of cases	MAF		OR (95% CI)	*P* value
		Cases	Controls		
Italians	96	0.005	0.002	4.08 (0.39-42.02)	.2
Spanish	101	0.040	0.005	9.53 (4.00-22.71)	3.5 × 10^−7^
Northern Europeans	17	0	0.009	—	—

OR and *P* value as defined in Table II.

**Table E7:** Effect size of rs71437970 with immediate hypersensitivity reactions broken down by drug classes and individual drugs

Drug	Ethnicity∗	No. of cases	Case MAF	OR (95% CI)	*P* value
Cephalosporins	Caucasian	162	0.05	0.64 (0.77-1.81)	.77
Cefaclor	Caucasian	23	0.09	0.58 (0.34-4.90)	.34
Cefazolin	Caucasian	17	0.06	0.34 (0.63-5.81)	.63
Cefotaxime	Italian	17	—	—	—
Ceftazidime	Italian	17	—	—	—
Ceftriaxone	Italian	48	—	—	—
Cefuroxime	Caucasian	14	0.05	0.18 (0.76-10.15)	.76
Penicillins	Caucasian	501	0.08	1.78 (1.36-2.32)	2.3 × 10^−5^
Amoxicillin	Caucasian	166	0.11	2.93 (2.05-4.19)	3.8 × 10^−9^
Ampicillin	Italian	29	0.02	0.29 (0.04-2.07)	.22
Co-amoxiclav	Caucasian	218	0.07	1.48 (0.99-2.21)	.06
Phenoxymethylpenicillin	Caucasian	25	0.02	1.59 (0.34-7.46)	.56
Piperacillin	Caucasian	18	0.11	2.13 (0.71-6,40)	.18
Bacampicillin	Italian	21	0.07	1.33 (0.39-4.51)	.65

OR and *P* value as defined in Table II.

∗ Ethnicity—Caucasian is applied to patients of Spanish, Italian and Northern European descent and confirmed by principle component analysis. Where only 1 nationality was available for a particular drug, this is indicated and only appropriate matching controls were chosen.

**Table E8:** Effect size of rs71437970 and amoxicillin-induced immediate hypersensitivity reactions across the different ethnic clusters

	No. of cases	Minor allele frequency		OR (95% CI)	*P* value
		Cases	Controls		
Italians	52	0.15	0.05	2.91 (1.59-5.32)	.0005
Spanish	80	0.09	0.04	2.55 (1.42-4.57)	.002
North Europeans	24	0.12	0.04	3.89 (1.54-9.86)	.004

OR and *P* value as defined in Table II.

## Methods

### Imputation procedure

Prephasing and imputation was performed in batches by dividing the cases and controls according to the genotyping platform. For each batch, we first prephased the genotype scaffold using SHAPEIT (version v2.r727).^E1^ Imputation was then undertaken using IMPUTE2 (version 3)^E2^ using the reference panel from the 1000 Genomes Project (release v3).^E1^ We used the “all ancestries” reference panel to improve the quality of the imputation especially of lower frequency variants.^E2^ For downstream analysis, we used best-guess genotypes retaining imputed genotypes with posterior probability >0.9. In the combined dataset, we then retained SNPs with: (1) no significant difference in missingness between cases and controls (*P* > .0001); (2) no significant deviation from Hardy-Weinberg equilibrium (*P* > .0001); (3) < 5% missing genotypes in all batches; (4) imputation information score >0.8 in all batches; and (5) MAF in the European descent haplotypes from the 1000 Genomes Project reference panel of at least 0.01. Finally, we compared SNP allele frequencies in controls between genotyping platforms. Specifically, we tested for association of genotyping platforms with SNPs in same ethnicity controls in a logistic regression framework, under an additive genetic model, with adjustment for principal components from smartPCA to account for population structure. SNPs with nominal evidence of association (*P* < .0001) were excluded. Sex chromosomes and mitochondria were not imputed.

For each batch, HLA alleles were inferred using HIBAG^E3^ using the reference predictor panels specific for the genotyping chip and Caucasian ancestry. Amino acid changes were inferred using SNP2HLA utilizing the reference data collected by the Type 1 Diabetes Genetics Consortium.^E4^
